# Analysis of hematological indicators via explainable artificial intelligence in the diagnosis of acute heart failure: a retrospective study

**DOI:** 10.3389/fmed.2024.1285067

**Published:** 2024-03-27

**Authors:** Rustem Yilmaz, Fatma Hilal Yagin, Cemil Colak, Kenan Toprak, Nagwan Abdel Samee, Noha F. Mahmoud, Amnah Ali Alshahrani

**Affiliations:** ^1^Department of Cardiology, Samsun Training and Research Hospital, Samsun University Faculty of Medicine, Samsun, Türkiye; ^2^Department of Biostatistics and Medical Informatics, Inonu University Faculty of Medicine, Malatya, Türkiye; ^3^Department of Cardiology, Faculty of Medicine, Harran University, Sanlıurfa, Türkiye; ^4^Department of Information Technology, College of Computer and Information Sciences, Princess Nourah bint Abdulrahman University, Riyadh, Saudi Arabia; ^5^Department of Rehabilitation Sciences, Health and Rehabilitation Sciences College, Princess Nourah bint Abdulrahman University, Riyadh, Saudi Arabia; ^6^Department of Computer Science, Applied College, Princess Nourah bint Abdulrahman University, Riyadh, Saudi Arabia

**Keywords:** acute heart failure, XGBoost, explainable artificial intelligence, SHAP, hematological parameters

## Abstract

**Introduction:**

Acute heart failure (AHF) is a serious medical problem that necessitates hospitalization and often results in death. Patients hospitalized in the emergency department (ED) should therefore receive an immediate diagnosis and treatment. Unfortunately, there is not yet a fast and accurate laboratory test for identifying AHF. The purpose of this research is to apply the principles of explainable artificial intelligence (XAI) to the analysis of hematological indicators for the diagnosis of AHF.

**Methods:**

In this retrospective analysis, 425 patients with AHF and 430 healthy individuals served as assessments. Patients’ demographic and hematological information was analyzed to diagnose AHF. Important risk variables for AHF diagnosis were identified using the Least Absolute Shrinkage and Selection Operator (LASSO) feature selection. To test the efficacy of the suggested prediction model, Extreme Gradient Boosting (XGBoost), a 10-fold cross-validation procedure was implemented. The area under the receiver operating characteristic curve (AUC), F1 score, Brier score, Positive Predictive Value (PPV), and Negative Predictive Value (NPV) were all computed to evaluate the model’s efficacy. Permutation-based analysis and SHAP were used to assess the importance and influence of the model’s incorporated risk factors.

**Results:**

White blood cell (WBC), monocytes, neutrophils, neutrophil-lymphocyte ratio (NLR), red cell distribution width-standard deviation (RDW-SD), RDW-coefficient of variation (RDW-CV), and platelet distribution width (PDW) values were significantly higher than the healthy group (*p* < 0.05). On the other hand, erythrocyte, hemoglobin, basophil, lymphocyte, mean platelet volume (MPV), platelet, hematocrit, mean erythrocyte hemoglobin (MCH), and procalcitonin (PCT) values were found to be significantly lower in AHF patients compared to healthy controls (*p* < 0.05). When XGBoost was used in conjunction with LASSO to diagnose AHF, the resulting model had an AUC of 87.9%, an F1 score of 87.4%, a Brier score of 0.036, and an F1 score of 87.4%. PDW, age, RDW-SD, and PLT were identified as the most crucial risk factors in differentiating AHF.

**Conclusion:**

The results of this study showed that XAI combined with ML could successfully diagnose AHF. SHAP descriptions show that advanced age, low platelet count, high RDW-SD, and PDW are the primary hematological parameters for the diagnosis of AHF.

## 1 Introduction

Heart failure (HF) is a significant global health concern, leading to substantial rates of illness and death. The prevalence of heart failure (HF) is on the rise in both developed and developing nations, primarily attributed to the extension of life expectancy. The leading factors contributing to this phenomenon are the escalating incidence of chronic ischemic heart diseases and hypertension. Therefore, healthcare costs are increasing day by day (1) and this situation negatively affects the quality of life of individuals. In the European Society of Cardiology (ESC) 2021 HF guidelines, HF is classified as preserved ejection fraction HF (HFpEF) (Left Ventricular Ejection Fraction (LVEF) > 50%), mildly reduced ejection fraction HF (HFmrEF) (LVEF <41–49%) and reduced ejection fraction HF (HFrEF) (LVEF <40%), respectively (2).

Acute heart failure (AHF) is characterized by the sudden or gradual signs of symptoms and indications associated with heart failure. The prevailing etiology of this condition is the abrupt deterioration of chronic heart failure. This clinical condition holds significant importance in the medical field as it necessitates hospitalization and has the potential to result in mortality. Hence, prompt initiation of diagnosis and treatment for patients admitted to the emergency department is imperative (3). In the diagnosis of AHF, brain natriuretic peptide (BNP) and N-terminal proBNP (NT-proBNP) are employed alongside electrocardiography (ECG), echocardiography (ECO), blood tests, as well as clinical signs and complaints. When the B-type natriuretic peptide (BNP) is not available, a chest X-ray of the pulmonary fields can be utilized. In the diagnosis of acute heart failure; NT-proBNP values are >450 pg./mL for <55 years old, >900 pg./mL for 55–75 years old, and > 1800 pg./mL for>75 years old, while BNP level below 100 pg./mL, and NT-proBNP level below 300 pg./mL are indication of the absence of acute heart failure (4–6). However, BNP and NT-proBNP values may be low in end-stage heart failure, acute pulmonary edema, and acute right heart failure. On the contrary, elevated levels of certain markers may not necessarily be indicative of the presence of acute heart failure. The prevalence of this condition is notably elevated in individuals diagnosed with chronic renal failure and atrial fibrillation (7).

Anemia is the most significant and independent driver of HF mortality, according to numerous studies that have used hematological markers to predict HF prognosis (8, 9). Furthermore, it has been observed that red-cell distribution width (RDW) is correlated with mortality, regardless of the presence of anemia (10–12). Previous research has indicated that elevated leukocyte levels and decreased lymphocyte counts are indicative of increased mortality risk in AHF (13). At present, there is a lack of a prompt and conclusive laboratory assay for the diagnosis of acute heart failure. The existing literature primarily focuses on the prognostic implications of hematological parameters.

Artificial intelligence (AI) plays a crucial role in numerous clinical decision support systems, facilitating the use of computational methods to make inferences that are comparable to human reasoning processes (14). The strategies presented in this context are founded upon medical information that has been either explicitly encoded or automatically generated from medical data using machine learning techniques. Explainable AI (XAI) has the potential to facilitate the prioritization of patients’ well-being and enable them to make independent and well-informed choices regarding their healthcare in conjunction with medical professionals (15). Building on the valuable information emphasized in various medical studies, the current research aims to analyze hematological markers to diagnose acute heart failure based on the implications of XAI. This study contributes to the state of the art by the following:

- Introducing a hybrid ML prediction model compromising LASSO, and XGBoost for rapid and accurate diagnosing AHF using hematological markers, offering a potential alternative to traditional diagnostic methods.
- Creating a dataset of 425 AHF patients and 430 healthy persons from Turkey to assess the feasibility and efficiency of the suggested model in a clinical environment.
- Utilizing XAI, particularly SHAP analysis, to evaluate the ML model’s predictions and emphasize key diagnostic parameters, improving transparency and interpretability.
- Identifying key risk variables for AHF diagnosis, such as platelet distribution width, age, and red cell distribution width-standard deviation, offers vital insights for clinicians and researchers.

The current work is organized as follows: Section 1 provides an overview of the subject, discusses previous studies, and summarizes the contributions. Section 2 covers the study design, dataset, and proposed technique. The results are presented in Section 3. Section 4 will discuss the attained results. Section 5 pertains to limitations, whereas section 6 provides the conclusion.

## 2 Materials and methods

### 2.1 Study design and dataset

In this observational study, patients who applied to our hospital due to acute heart failure between January 2020 and April 2023 were retrospectively analyzed. Patients with AHF were divided into three groups according to their left ventricular ejection fraction: HFpEF (LVEF >50%), HFmrEF (LVEF <41–49%), and HFrEF (LVEF <40%) as specified by transthoracic echocardiography. The inclusion criteria in the current study were as follows: 425 patients older than 18 years hospitalized for worsening HF with two or more signs or symptoms of fluid retention (e.g., dyspnea, paroxysmal nocturnal dyspnea, orthopnea, ankle edema, or jugular venous distension) and 430 healthy control group, individuals without HF (normal cardiac examination and echocardiography findings) who applied to the cardiology department due to nonspecific cardiac complaints such as weakness, fatigue, and palpitations. With G*Power software (University of Dusseldorf, Dusseldorf, Germany, version 3.0.1), the independent sample t-test was used to calculate sample size and actual power (α = 0.05, power = 0.80, effect size = 0.35). The results revealed that with a sample size of 260 participants, the actual power was 80.2% (16).

The exclusion criteria from the study were as follows: Patients with hematological malignancies; those who use drugs known to affect the complete blood count (e.g., chemotherapy drugs that suppress the bone marrow, overdose of warfarin); those with a hemoglobin value of less than 10 g/dL, those with active bleeding, those with acute infections, those with acute myocardial infarction, severe renal impairment (estimated glomerular filtration rate (eGFR) <15 mL/min/1.73 m^2^), severe liver impairment or with chronic obstructive pulmonary disease; those with chronic inflammatory diseases (in case of acute exacerbation of the disease). The study was conducted by the Declaration of Helsinki, and approved by the Samsun University Clinical Research Ethics Committee (protocol code 2023/10/10 and 24.05.2023).

Age, gender, underlying diseases, LVEF percentage, and clinical and echocardiographic characteristics were obtained from the medical records of the patients and the control group at the first admission to the hospital. At the same time, the hematological and biochemical laboratory results obtained from the venous blood taken at the first application to the cardiology department of both groups; urea, creatinine, c-reactive protein (CRP), aspartate transaminase (AST), alkaline phosphatase (ALT), hemoglobin (Hbg) values, hematocrit (Hct) values, mean corpuscular volume (MCV) values, mean corpuscular hemoglobin (MCH) values, MCH concentration (MCHC) values, red-cell distribution width-standard deviation (RDW-SD) values, RDW-coefficient of variation (RDW-CV) values, mean platelet volume (MPV) values, platelet width of distribution (PDW) values, procalcitonin (PCT) values, erythrocyte (RBC) platelet (PLT) counts and white blood cell (WBC), neutrophil (NEU), lymphocyte (LY), neutrophil-lymphocyte ratio (NLR), basophil (BA), monocytes (MO), and eosinophil (EO) counts were reached (The dataset is available from Supplementary materials).

### 2.2 Statistical analysis

The conformity of the variables to the normal distribution was examined by visual (histogram and probability graphs) and analytical (Shapiro–Wilk Test) methods. The assumption of homogeneity of variances was examined with the Levene test. Descriptive statistics are expressed as median, interquartile range for non-normally distributed variables, and mean ± standard deviation for normally distributed variables. Independent Samples *t*-test was used in the comparison of the variables that met the parametric test assumptions of the two groups. The Mann–Whitney U test was used to compare the two groups in terms of variables that did not meet the parametric test assumptions. Frequency (*n*) and percentage (%) values were calculated for the qualitative variables, and the relationships between the two qualitative variables were examined using the Chi-square test. A *p*-value of <0.05 was considered statistically significant in all results. Statistical analyses were performed using the SPSS 28.0 (IBM Corp., Armonk, NY, United States) package program.

### 2.3 ML and XAI approach

The LASSO feature selection algorithm was employed in the research to ascertain the primary risk factors associated with Acute Heart Failure. Following the implementation of LASSO, a predictive model was constructed utilizing the variables that were chosen during the selection process (17). The utilization of the XGBoost algorithm, renowned for its good performance, scalability, and adaptability, was employed in the diagnosis of Acute Heart Failure. XGBoost is widely acknowledged as a robust algorithm for handling structured data and employs a boosting technique that progressively incorporates new models derived from the collective knowledge of the community. During each iteration, the algorithm assesses the performance of the current models and proceeds to train a new model with the objective of minimizing errors made by the ensemble (18, 19). The XGBoost algorithm’s capacity to be scalable in all circumstances and quick processing execution are its key accomplishments. The XGBoost eliminates overfitting concerns and takes into account the bias-variance trade-off by offering bagging-bootstrap aggregation and feature randomness (20–23).

The rationale behind selecting XGBoost and SHAP for diagnosing acute heart failure based on hematological indicators is as follows:

1. *XGBoost*: XGBoost is a powerful and widely used machine learning algorithm known for its efficiency and effectiveness in classification tasks, such as diagnosing medical conditions, due to its ability to handle complex relationships between input variables and predict outcomes accurately. Lately, the XGBoost has been employed in the diagnosis of heart diseases and achieved excellent performance (24–27). Doki et al. have introduced a straightforward and efficient diagnostic method that utilizes XGBoost with a feature selection algorithm for predicting heart disease in datasets with limited records. Budholiya et al. have introduced a diagnostic system that utilizes the XGBoost classifier to predict heart disease and it has been shown to outperform other classical ML methods such as the Random Forest and Extra Tree classifiers. Tian et al. introduced the “All-in” XGBoost model for diagnosing heart failure and it attained the best performance (24–27).
2. *SHAP*: SHAP is a method for explaining individual predictions of machine learning models. It provides insights into the contribution of each input variable to the model’s predictions, allowing for a better understanding of the model’s decision-making process. In the context of medical diagnosis, SHAP can help identify which hematological indicators are most influential in predicting acute heart failure such as the work recently done in (28–30).

By leveraging the strengths of XGBoost for predictive modeling and SHAP for interpretability, this study aims to develop a robust and explainable diagnostic model for acute heart failure based on hematological parameters. This combination allows for accurate predictions while also providing insights into the underlying biological mechanisms and clinical relevance of the predictive features.

The validation method employed in this study was 10-fold cross-validation. The model’s performance was assessed using several evaluation metrics, including Accuracy, PPV, NPV, F1-score, Area Under the Curve (AUC), and the Receiver Operating Characteristic (ROC) curve was plotted. The Brier score was computed in order to assess the calibration of the model. The Brier score is a metric that quantifies the calibration of a model, with values ranging from 0 to 1. A lower Brier score indicates a higher level of calibration, indicating a better performance of the model (31). The importance of the variables incorporated in the model was assessed using both permutation-based importance and SHAP, which is an explainable artificial intelligence (XAI) technique (32). SHAP is a method that can work effectively with both model-independent and model-specific annotations. SHAP calculates the Shapley values for each feature and uses them to determine the importance of the feature. SHAP is an excellent tool for evaluating fidelity, reliable, and detailed descriptions for classification and prediction models (33). Individual aggregated local SHAP values can be used for general explanations due to their additive properties. For deeper ML approaches such as fairness, pattern tracking, and cohort analysis, SHAP can provide a better interpretation (34).

## 3 Results

In this section, a comprehensive evaluation of the statistical significance of the collected dataset, ensuring the reliability and validity of the data used in the study. The performance metrics of the developed prediction model for diagnosing AHF. Key performance indicators such as accuracy, sensitivity, specificity, area under the receiver operating characteristic curve (AUC-ROC), and F1 score have been listed to quantify the model’s effectiveness in distinguishing between AHF patients and healthy individuals. An interpretation of the decision of the introduced ML model is presented by explaining the shapely graphs. The SHAP values for each feature are visualized, which allows us to shed light on the underlying mechanisms and relationships within the data. This allows us to highlight how particular attributes contribute to the predictions made by the model.

A total of 855 people were retrospectively included in the study, of which 430 (50.3%) were healthy controls and 425 (49.7%) were patients with Acute Heart Failure between January 2020 and April 2023. Of the patients with AHF, 392 (92.2%) had an HFrEF, 18 (4.3%) had an HFmrEF, and 15 (3.5%) had an HFpEF. All of the healthy group, 430 (100) had an LVEF of 50% and above. Of the participants, 369 (43.2%) were female and 486 (56.8%) were male.

Table 1 presents the descriptive statistics of the sociodemographic data of the patients. Males had more Acute Heart Failure than females (*p* < 0.001) and patients were significantly older than controls (*p* < 0.001).

**Table 1:** Descriptive statistics of the variables with respect to the study groups.

Variable		Group		*p*-value
		Control	Acute heart failure	
Sex*	Female	220 (51.16%)	149 (35.06%)	<0.001
	Male	210 (48.84%)	276 (64.94%)	
Age**		58.35 ± 11.28	64.21 ± 9.96	<0.001

*The variable was summarized as *n* (%); **The variable is summarized as mean ± standard deviation.

Descriptive statistics for complete blood count parameters in acute heart failure patients and healthy controls are presented in Table 2. According to Table 2, the results of WBC, MO, NEU, NLR, RDW-SD, RDW-CV and PDW increased significantly in patients with acute heart failure (*p* < 0.05). RBC, HGB, BA, LY, MPV, PLT, HCT, MCH, and PCT results were found to be significantly lower in the patient group compared to healthy controls (*p* < 0.05).

**Table 2:** Statistics on complete blood count parameters.

Variable*	Group		*p*-value
	Control	Acute heart failure	
WBC (10^9/L^)	7.49 (2.325)	8.19 (2.79)	<0.001
RBC (10^12/L^)	4.695 (0.68)	4.59 (0.75)	0.008
HGB (g/dL)	13.65 (2.1)	13.2 (2.2)	<0.001
BA (10^9/L^)	0.04 (0.04)	0.03 (0.08)	0.041
EO (10^9/L^)	0.14 (0.13)	0.13 (0.15)	0.766
LY (10^9/L^)	2.29 (0.92)	1.87 (1.13)	<0.001
MO (10^9/L^)	0.56 (0.22)	0.64 (0.3)	<0.001
NEU (10^9/L^)	4.295 (1.758)	5.13 (2.5)	<0.001
NLR	1.802 (0.941)	2.7 (2.288)	<0.001
MPV (fL)	10.05 (1.3)	9.69 (2.2)	<0.001
PLT (10^9/L^)	256 (84)	241 (78)	<0.001
HCT (%)	40.9 (5.6)	39.79 (7)	<0.001
RDW-SD	41 (4.8)	43.9 (6.4)	<0.001
RDW-CV	13.2 (1.3)	14.6 (2.69)	<0.001
MCH (pg)	29.1 (2.475)	28.8 (2.9)	0.029
MCHC (g/dL)	33.2 (2)	33.29 (1.9)	0.525
PDW (fL)	12 (3.475)	16.39 (3.1)	<0.001
PCT (%)	0.25 (0.07)	0.22 (0.08)	<0.001

Variables were summarized as median (interquartile range); WBC, leukocyte; RBC, erythrocyte; HGB, hemoglobin; BA, basophil; EO, eosinophil; LY, lymphocyte; MO, monocyte; NEU, neutrophil; NLR, neutrophil/lymphocyte; MPV, mean platelet volume; PLT, platelet; HCT, hematocrit; RDW-SD, red cell distribution width-standard deviation; RDW-CV, Red cell distribution width-coefficient of variation; MCH, mean erythrocyte hemoglobin; MCHC, mean corpuscular hemoglobin concentration; PDW, platelet distribution width; PCT, procalcitonin test. *Mann Whitney U test.

**Figure 1:**
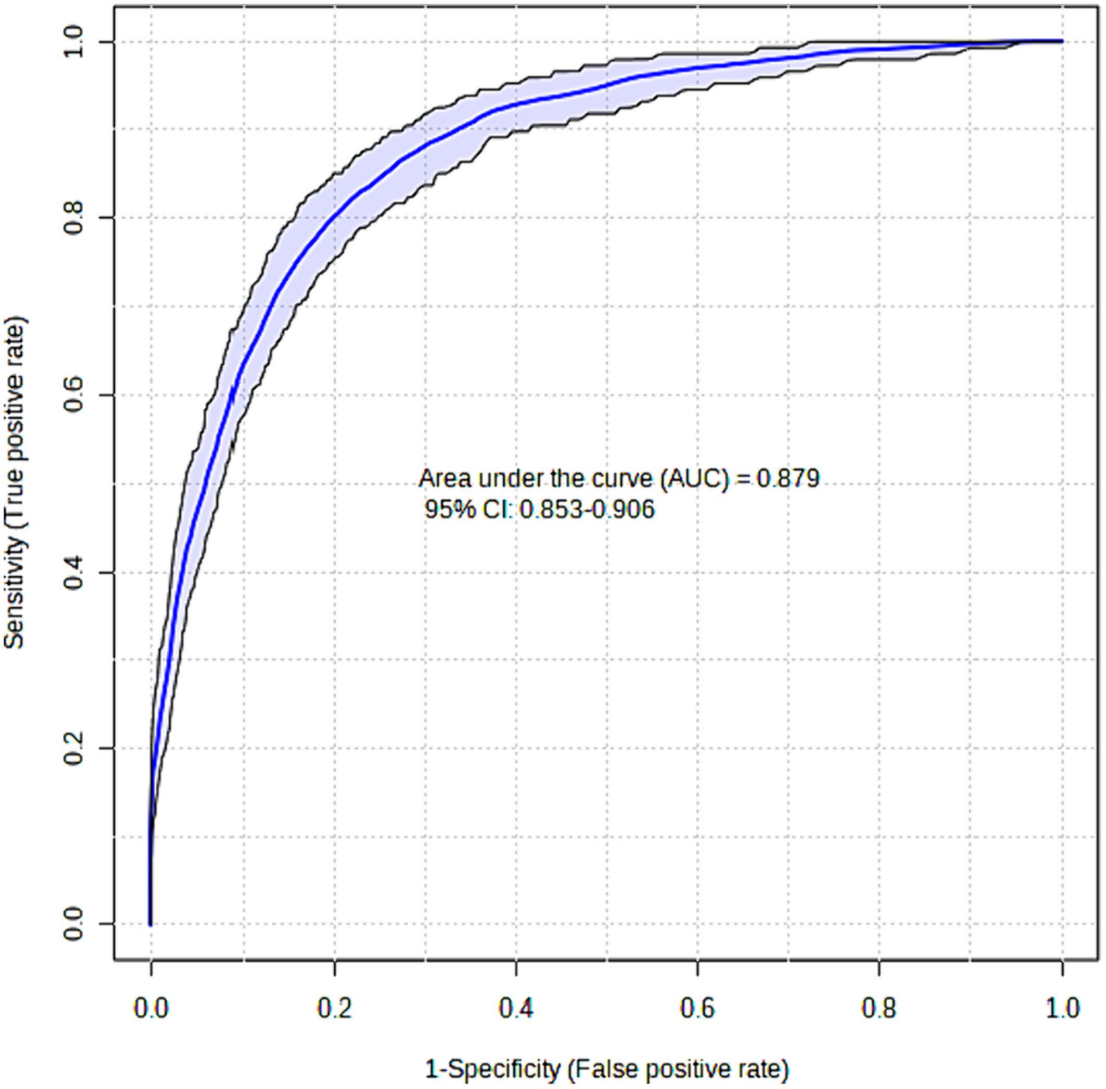
ROC curve for the XGBoost model.

The XGBoost model that was proposed demonstrated a high level of success in accurately predicting instances of Acute Heart Failure. The XGBoost prediction model achieved an accuracy of 87.5%, an F1-score of 87.4%, and an AUC value of 87.9%. The Brier score was computed to assess the calibration of the model. A model is considered to be well-calibrated when its Brier score approaches zero. Based on the Brier score, the XGBoost model demonstrated a high level of quality, with a value of 0.036 (refer to Table 3; Figure 1).

**Table 3:** Performance measures for the prediction of acute heart failure of the XGBoost model.

Metrics	Value
Accuracy	0.875
PPV	0.877
NPV	0.873
F1-score	0.874
AUC	0.879
Brier-score	0.036

PPV, positive predictive value; NPV, negative predictive value; AUC, Area under the ROC curve.

**Figure 2:**
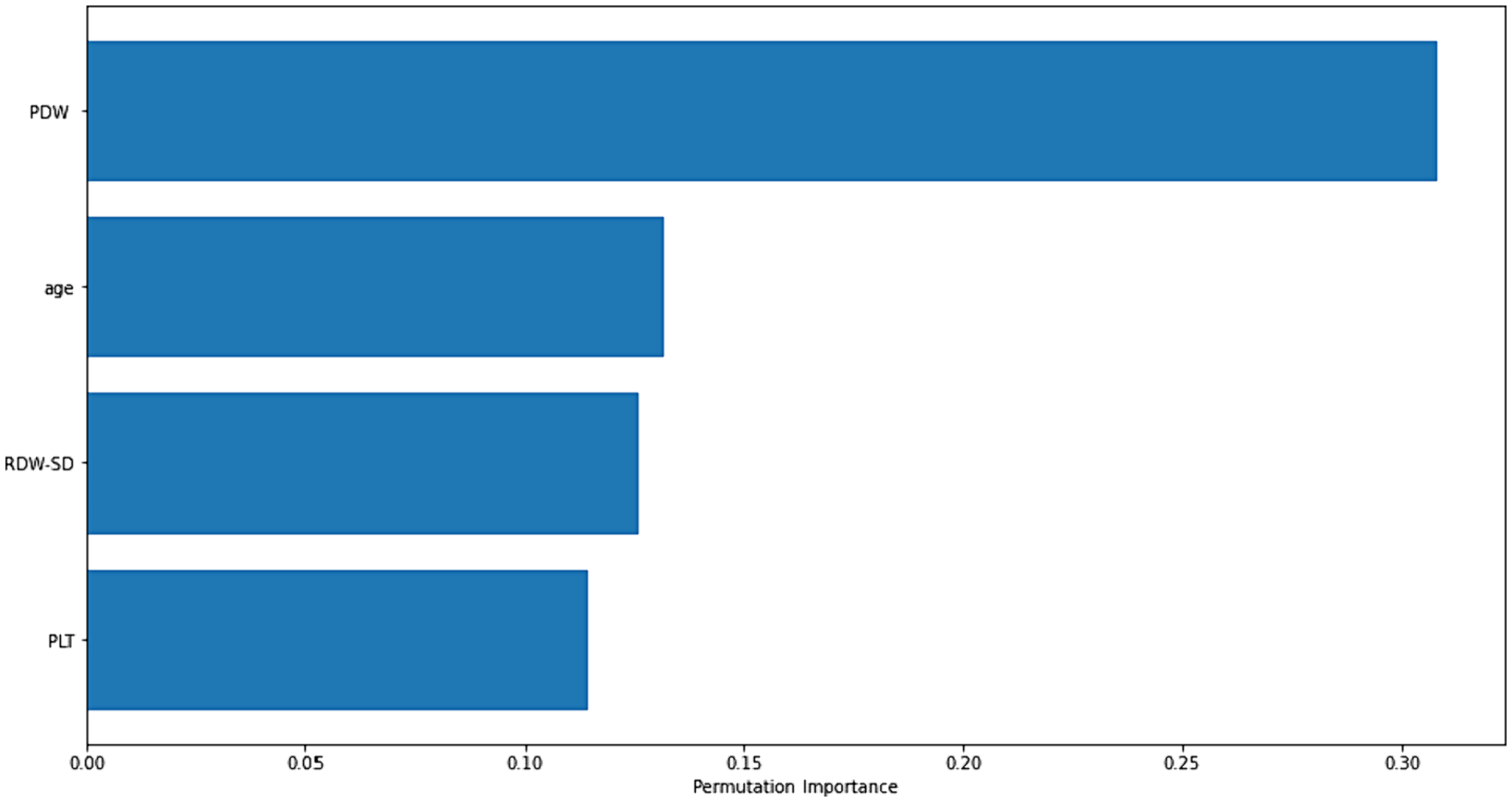
Permutation-based importance plot of the most important risk factors for diagnosing acute heart failure.

Figure 2 displays the importance graphs of the variables incorporated in the XGBoost model, which have been identified as important risk factors through the utilization of the Lasso feature selection method. The Lasso method successfully identified several key risk factors, including PDW, age, RDW-SD, and PLT, that play a significant role in distinguishing cases of Acute Heart Failure. The findings particularly demonstrated the significance of platelet distribution width (PDW) in distinguishing the disease.

**Figure 3:**
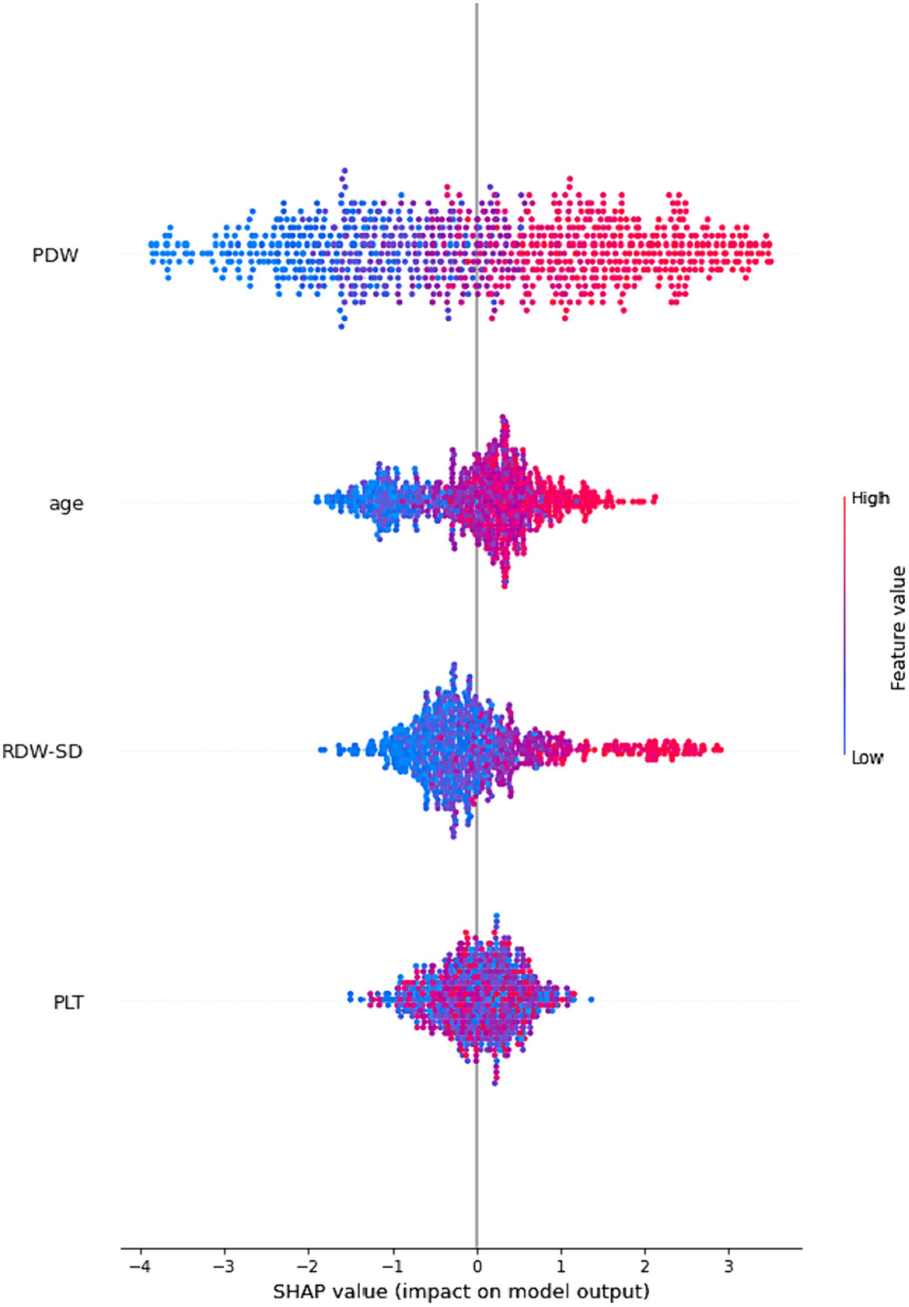
An illustration of the interpretability of the XGBoost model. The four most significant risk factors are presented in a descending order of importance based on their respective SHAP values. A higher SHAP value indicates a greater probability of a patient experiencing acute heart failure.

Figure 3 employs global SHAP values, which depict the positive or negative impact of biomarker candidates on the XGBoost model’s prediction. This visual representation serves to highlight the importance of biomarker candidates in influencing model decisions. A positive SHAP value indicates a positive contribution to the target variable, while a negative SHAP value indicates a negative contribution. Furthermore, the data points on the graph are shaded based on the normalized values of the variables. The value of the variable increases as it approaches the color pink and decreases as it approaches the color blue. Consequently, it was found that older age is associated with an elevated risk of Acute Heart Failure, as well as higher values of Platelet Distribution Width (PDW) and Red Cell Distribution Width-Standard Deviation (RDW-SD).

## 4 Discussion

With an emphasis on the use of artificial intelligence (AI) for the precise and quick diagnosis of AHF, this section provides a critical evaluation of our study’s results and places them in the context of the current literature. The importance, relevance, and possible directions for further study in this field are our goals. We explore the mechanics, clinical relevance, and wider implications of our study’s findings through a thorough evaluation and synthesis of the results. The purpose of this study is to shed light on how AI can improve healthcare practices and patient outcomes by investigating the link between AI and AHF diagnosis. We also go over the study’s limitations and provide some ideas for where the field could go from here in terms of research to help overcome them and move it forward.

Artificial intelligence enhances the quality of healthcare services by enabling more precise, rapid, and personalized diagnosis, treatment, and patient care in the medical field. The utilization of artificial intelligence in analyzing vast medical data and predicting diseases improves early diagnosis and subsequently facilitates more effective management of treatment processes, leading to an enhancement in patients’ quality of life. Furthermore, artificial intelligence plays a significant role in accelerating scientific discoveries and advancing the medical field in areas such as medical image analysis, genetic research, and drug development.

This study used biostatistical analysis and an ML model combined with XAI to investigate potential hematological indicators for the diagnosis of AHF. The observed results indicated that the WBC, MO, NEU, NLR, RDW-SD, RDW-CV, and PDW values of patients with AHF were statistically higher than those of the healthy group (*p* < 0.05). However, compared to healthy controls, AHF patients had significantly reduced RBC, HGB, BA, LY, MPV, PLT, HCT, MCH, and PCT levels (*p* < 0.05). PDW, age, RDW-SD, and PLT were found to be the most relevant hematological indicators for differentiating AHF by one of the machine learning models using the Lasso approach. The results revealed that especially PDW may be important in differentiating the disease in question. In this study, the proposed XGBoost model performed quite successfully for the diagnosis of AHF. With the XGBoost prediction model, 87.5% accuracy, 87.4% F1-score, and 87.9% AUC value were obtained. The XGBoost model as important risk factors by the Lasso feature selection method are presented.

In a study conducted in the literature, hemoglobin, serum creatinine, LDL (Low-density lipoprotein) cholesterol, HDL (High-density lipoprotein) cholesterol, triglycerides, ALT, AST, high-sensitive cardiac troponin I, and C-reactive protein (CRP) results of 59 patients with heart failure and 108 patients with chronic ischemic heart disease were evaluated. A predictive model based on logistic regression was created in the study, which aimed to identify important independent markers of the outcome of heart failure versus chronic-ischemic heart disease. The authors obtained 80.5% AUC with the model (Hb + serum creatinine + AST + hs-cTnI + CRP) created to differentiate between heart failure and chronic ischemic heart disease (35). In our current study, we differentiated AHF with an AUC of 87.9% using a larger sample based on hematological parameters. Ahmad et al. investigated the strong link between several parameters and the incidence of heart failure in the field of heart failure (36). Only serum creatinine and left ventricular ejection fraction (LVEF), according to Chicco and Jurman, may be used to predict the survival rate of patients with heart failure (37). To save the standard of care in the hospital system, Sohrabi et al. used classification algorithms (i.e., DT, Artificial Neural Networks (ANN), SVM, and LR) and employed AUC and accuracy as assessment indicators (38). To predict a patient’s risk of developing heart failure, Lafta et al. built a neural network classifier using the global weight of attribute contribution (39). The findings demonstrated that the technique was capable of precisely predicting the clinical risk of heart failure. Yang et al. (33) presented an SVM-based scoring model for the HF diagnosis. They used it on 289 different samples of clinical data that were gathered from Zhejiang Hospital. Three groups were created from the sample: the HF group, the HF-prone group, and the healthy group. The accuracy of their HF diagnosis was 74.44% overall, which was a significant increase over earlier studies which they matched their findings. Accuracy reaches 87.5%, especially in the HF-prone group, which suggests that the suggested methodology is workable for HF early detection (40). Although the AHF classification performance was partially good in most of these studies, XAI was not examined with model calibration. In addition to the successful estimation of AHF in the current study, calibrating the model and interpreting the results with XAI led to more reliable clinical results for AHF and the creation of an explainable model.

Platelets are cellular fragments that serve a crucial function in the processes of blood coagulation and wound healing (41–43). Prior research has indicated that an unexplained decrease in platelet count is linked to mortality in acute heart failure, particularly among individuals with advanced-stage cardiac dysfunction (44–46). The etiology of thrombocytopenia in individuals with heart failure remains elusive. Several theories have been proposed about this matter. Examples of physiological conditions that can occur include dysfunction of bone marrow, reduced blood flow, and programmed cell death (47). In the present investigation, the descriptive statistics findings indicate a statistically significant decrease in platelet count among patients diagnosed with AHF compared to the control group of individuals in good health (*p* < 0.05). Furthermore, the artificial intelligence models employed in our present investigation have demonstrated that a diminished count of platelets serves as a significant hematological parameter for the diagnosis of acute heart failure. The potential cause of the decreased platelet count observed in patients with AHF in comparison to the healthy control group could be attributed to two factors: the reduction in platelet lifespan resulting from hypervolemia, and the impairment of platelet structure due to compromised blood oxygenation, potentially leading to apoptosis. This can potentially lead to hemorrhage and result in the development of anemia. The presence of this condition may lead to unfavorable outcomes in individuals affected by it. Nevertheless, the available information on this subject is insufficient. Further research and investigation are required to address this topic.

In developed as well as developing countries, where there has been a notable rise in average life expectancy, heart failure has emerged as a significant factor contributing to hospitalizations. Heart failure is a condition that is influenced by various factors, including hypertension, chronic ischemic heart disease, diabetes mellitus, and obesity. These causative factors have been observed to exhibit an increased prevalence with advancing age, thereby contributing to the higher incidence of heart failure. This phenomenon results in significant rates of both mortality and morbidity (1). In the present investigation, it was observed that the average age of individuals who sought medical attention from the cardiology department for acute heart failure was significantly greater compared to the control group (*p* < 0.05). Furthermore, within the context of artificial intelligence modeling, age has been identified as a significant parameter in the diagnosis of acute heart failure (AHF). The incidence of heart failure with preserved ejection fraction, a condition that is more prevalent among females, demonstrates an upward trend as individuals advance in age (48, 49). When kidney functions decline due to the natural aging process, there is a potential for acute heart failure to occur as a result of both increased mortality related to heart failure and reduced urinary excretion. This phenomenon has the potential to result in a higher incidence of hospital emergency department admissions for acute heart failure.

Red-cell Distribution Width (RDW) is a laboratory test that measures the heterogeneity of the size distribution of erythrocytes. Previous research has demonstrated that elevated RDW values serve as a significant predictor of mortality in individuals diagnosed with heart failure (11). In another study, patients with HF may have elevated RDW due to secondary causes. The main secondary causes are renal failure, malnutrition, chronic inflammation, and inadequate erythropoiesis. In patients with HF, causes such as renal hypoperfusion due to low cardiac output, the effect of diuretics used, and comorbidities (for example, diabetes mellitus, hypertension, atherosclerotic vascular diseases) may cause chronic renal failure. This can cause both anemia and high RDW by reducing erythropoietin production. In addition, malnutrition in heart failure patients can cause iron deficiency anemia. In this case, it can indirectly increase the RDW value (50, 51). In our current study, according to the descriptive statistics results, RDW-SD was found to be significantly higher in patients with AHF than in the healthy control group (*p* < 0.05). In addition, artificial intelligence models used in our current study showed that RDW-SD is one of the important hematological parameter in diagnosing AHF. In our study, patients with a hemoglobin value of less than 10 g/dL, a high CRP, and a creatinine value above 2 mg/dL were not included in the study. AHF patients may have dilutional anemia due to hypervolemia. Intravascular volume increase may indirectly cause RDW-SD elevation. However, there is not enough information on this subject. More work needs to be done on this.

The Platelet Distribution Width is a laboratory assay utilized to quantify the degree of variability in platelet size distribution (52). Several studies have indicated an increased presence of PDW in individuals diagnosed with malignancies, cardiovascular disease, diabetes mellitus, and respiratory diseases (53–60). Furthermore, certain studies have demonstrated a positive correlation between elevated platelet distribution width and heightened rates of mortality and morbidity among individuals diagnosed with ischemic heart diseases, pulmonary thromboembolism, and advanced cancer (61–64). Nevertheless, the precise etiology of elevated PDW in these pathological conditions remains unclear. A recent study has revealed that oxidative stress has a detrimental effect on platelet functions. Furthermore, certain enzymes, such as chemokines and cytokines, which are elevated in patients with high platelet distribution width, have been found to enhance platelet activation. These enzymes promote the release of substances that initiate the process of clot formation from platelets. Other studies in the literature have established that this particular circumstance is associated with an elevated susceptibility to cardiovascular thrombosis, as well as an augmented 90-day morbidity and mortality rates (58–70). There is a lack of prior research examining the correlation between elevated PDW levels and their predictive and prognostic value in patients with Acute Heart Failure. Based on the findings of our present investigation, the descriptive statistics indicate a statistically significant elevation in PDW among individuals diagnosed with AHF compared to the healthy control cohort (*p* < 0.05). Furthermore, the artificial intelligence models employed in our present investigation have demonstrated that PDW is the most hematological parameter for diagnosing acute heart failure. In the context of acute heart failure, the hypervolemic condition, particularly observed in cases of total heart failure or right heart failure, can lead to spleen congestion. This congestion has the potential to disrupt the structure of platelets, resulting in an elevation of the Platelet Distribution Width value. Furthermore, it is worth noting that the hypervolemic condition has the potential to elevate the values of PDW and Red Cell Distribution Width due to the induction of intracellular edema in both erythrocytes and platelets. Consequently, this leads to a decline in the oxygen-carrying capability of compromised erythrocytes and an escalation in tissue hypoxia, thereby initiating a detrimental cycle. This phenomenon has the potential to exacerbate the prognosis in individuals diagnosed with heart failure. Similarly, the degradation of platelets can lead to both bleeding and thrombosis. Nevertheless, a dearth of information on this subject has persisted until the present day. Therefore, it is imperative to conduct thorough clinical research to gain a deeper understanding of this matter.

The findings of our investigation reveal that the integration of XAI with ML techniques holds significant promise for the accurate diagnosis of AHF. Through the application of SHAP values, we have identified that certain hematological parameters, namely advanced age, low platelet count, high Red Cell Distribution Width-Standard Deviation, and Platelet Distribution Width, play a pivotal role in diagnosing AHF. These insights underscore the utility of a Complete Blood Count as not only a readily accessible but also a cost-effective diagnostic tool. Given the simplicity and affordability of CBC, it stands out as a particularly valuable method for early detection and diagnosis in clinical settings.

The implications of our findings suggest that medical practitioners and researchers should place a heightened focus on these identified hematological parameters when evaluating individuals presenting symptoms of AHF. By doing so, it may be possible to enhance diagnostic accuracy and facilitate early intervention, potentially improving patient outcomes. Furthermore, our study advocates for the broader application of XAI within the field of cardiology research. The use of XAI can offer deeper insights and a more nuanced understanding of risk factors associated with heart diseases, including AHF. By leveraging XAI, researchers and clinicians can uncover more precise and individualized risk factors, thereby advancing the precision medicine approach in cardiology. The integration of XAI and ML presents a novel and effective approach to the diagnosis of AHF, highlighting the importance of specific hematological parameters obtained from CBC. Our study not only reinforces the value of CBC as a diagnostic tool but also opens new avenues for research in cardiology, emphasizing the potential of XAI to revolutionize the identification and management of risk factors in heart disease. We strongly recommend the adoption of XAI in cardiology research to further refine our understanding of AHF and to pave the way for more targeted and effective treatment strategies.

## 5 Comparing the performance with existing state of the art

We compared our proposed strategy with the current state of the art by analyzing previously published studies that have dealt with similar research problems or used similar datasets. We assessed the efficacy of our method in diagnosing acute heart failure by analyzing important parameters like accuracy, and AUC. The current study identified key risk variables for AHF diagnosis, such as platelet distribution width, age, and red cell distribution width-standard deviation, which offers vital insights for clinicians and researchers. Another paper assessed hematological predictors via XAI in the prediction of acute myocardial infarction and reported that accessible hematological parameters could enable medical personnel to make more informed decisions and give better treatment to a wider group of patients (71). In the relevant study, 83% accuracy and 91% AUC were obtained with the XGBoost model to distinguish AMI patients. When the results were examined, the findings of the current study were better.

## 6 Limitations

The introduced study has some limitations. First, our data source included patients from only one geographic region of Turkey, which limits generalizability and requires validation in other populations. Second, we used only hematological predictive variables in our ML approaches. Therefore, there is a need for future studies that present ML approaches that can extract different clinical risk factors and unstructured information such as clinicians’ free text notes. Third, although we examined a relatively large sample in AHF, we did not have the external validation set for the prediction model. Finally, although XAI and ML techniques provide valuable insights into the diagnosis of AHF based on hematological parameters, they may overlook important information related to patients’ medical history. The comprehension and integration of features that are required to be analyzed and to make an accurate and efficient diagnosis of AHF and the evaluation of therapeutic regimens are quite challenging and complicated tasks (72). Even though there has been significant progress in understanding the complex pathophysiology of HF, numerous challenges and complications still exist. To assist general practitioners in diagnosing heart failure at an earlier stage and in providing better follow-up for patients, it is necessary to develop an algorithm that incorporates the key features (history, clinical parameters, and anamnesis) that can be associated with heart failure (73). Incorporating additional human-oriented information, such as medical history, could enhance the accuracy and completeness of the diagnostic process. Therefore, further exploration of the connection between biological indicators and patient-specific medical history can enhance the applicability, and reliability of the introduced ML model.

## 7 Conclusion

The results of this study showed that XAI combined with ML could successfully diagnose AHF. SHAP descriptions show that advanced age, low platelet count, high RDW-SD, and PDW are the primary hematological parameters for the diagnosis of AHF. Complete blood count (CBC) is an easily available and cost-effective diagnostic technique. It may be advisable to direct attention to these parameters in individuals with AHF symptoms. In addition, we recommend the use of XAI in research related to the cardiology discipline, to distinguish more precise risk factors.

## Data availability statement

The datasets presented in this study can be found in online repositories. The names of the repository/repositories and accession number(s) can be found in the article/Supplementary material.

## Ethics statement

The study was conducted in accordance with the Declaration of Helsinki, and approved by the Samsun University Clinical Research Ethics Committee (protocol code 2023/10/10 and 24.05.2023). The studies were conducted in accordance with the local legislation and institutional requirements. The participants provided their written informed consent to participate in this study. Written informed consent was obtained from the individual(s) for the publication of any potentially identifiable images or data included in this article.

## Author contributions

RY: Writing – review & editing, Writing – original draft, Visualization, Validation, Supervision, Methodology, Investigation, Data curation, Conceptualization. FY: Writing – review & editing, Writing – original draft, Visualization, Validation, Supervision, Software, Resources, Methodology, Investigation, Formal analysis, Conceptualization. CC: Writing – review & editing, Writing – original draft, Visualization, Validation, Supervision, Resources, Methodology, Investigation, Data curation, Conceptualization. KT: Writing – review & editing, Writing – original draft. NA: Writing – review & editing, Writing – original draft, Validation, Resources. NM: Writing – review & editing, Writing – original draft, Visualization, Resources, Funding acquisition. AA: Funding acquisition, Resources, Writing – review & editing, Writing – original draft, Visualization.
