# Corrigendum: Analysis of hematological indicators via explainable artificial intelligence in the diagnosis of acute heart failure: a retrospective study

**DOI:** 10.3389/fmed.2024.1431299

**Published:** 2024-06-10

**Authors:** Rustem Yilmaz, Fatma Hilal Yagin, Cemil Colak, Kenan Toprak, Nagwan Abdel Samee, Noha F. Mahmoud, Amnah Ali Alshahrani

**Affiliations:** ^1^Department of Cardiology, Samsun Training and Research Hospital, Samsun University Faculty of Medicine, Samsun, Türkiye; ^2^Department of Biostatistics and Medical Informatics, Inonu University Faculty of Medicine, Malatya, Türkiye; ^3^Department of Cardiology, Faculty of Medicine, Harran University, Sanliurfa, Türkiye; ^4^Department of Information Technology, College of Computer and Information Sciences, Princess Nourah bint Abdulrahman University, Riyadh, Saudi Arabia; ^5^Department of Rehabilitation Sciences, Health and Rehabilitation Sciences College, Princess Nourah bint Abdulrahman University, Riyadh, Saudi Arabia; ^6^Department of Computer Science, Applied College, Princess Nourah bint Abdulrahman University, Riyadh, Saudi Arabia

**Keywords:** acute heart failure, XGBoost, explainable artificial intelligence, SHAP, hematological parameters

In the published article, there was an error in affiliation 6. Instead of “Department of Computer Science and Information Technology, Applied College, Princess Nourah bint Abdulrahman University, Riyadh, Saudi Arabia]”, it should be “Department of Computer Science, Applied College, Princess Nourah bint Abdulrahman University, Riyadh, Saudi Arabia”.

The authors apologize for this error and state that this does not change the scientific conclusions of the article in any way. The original article has been updated.

In the published article, there was an error in the Funding statement. It originally stated: “The author(s) declare that financial support was received for the research, authorship, and/or publication of this article. This study was supported by Princess Nourah bint Abdulrahman University, Riyadh, Saudi Arabia (Project Number PNURSP2024R732”. The correct Funding statement appears below.

